# Crowned dens syndrome: a rare differential diagnosis of meningitis

**DOI:** 10.1007/s43678-024-00850-w

**Published:** 2025-01-05

**Authors:** Andriko Palmowski, Jan Riedel, Paul Kamieniarz, Hildrun Haibel, Lorenz Bartsch, Eva Diehl-Wiesenecker

**Affiliations:** 1 Department of Emergency Medicine, Charité—Universitätsmedizin Berlin, Corporate Member of Freie Universität Berlin and Humboldt-Universität Zu Berlin, Campus Benjamin Franklin, 12203 Berlin, Germany; 2 Department of Rheumatology and Clinical Immunology, Charité Campus Mitte, Charité—Universitätsmedizin, Corporate Member of Freie Universität Berlin and Humboldt-Universität Zu Berlin, 10117 Berlin, Germany; 3 Section for Biostatistics and Evidence-Based Research, The Parker Institute, Bispebjerg Og Frederiksberg Hospital, University of Copenhagen, 2000 Frederiksberg, Denmark; 4 Department of Radiology, Charité—Universitätsmedizin Berlin, Corporate Member of Freie Universität Berlin and Humboldt-Universität Zu Berlin, 10117 Berlin, Germany; 5 Department of Gastroenterology, Infectiology and Rheumatology, Charité—Universitätsmedizin Berlin, Corporate Member of Freie Universität Berlin and Humboldt-Universität Zu Berlin, Campus Benjamin Franklin, 12203 Berlin, Germany

**Fig. 1:**
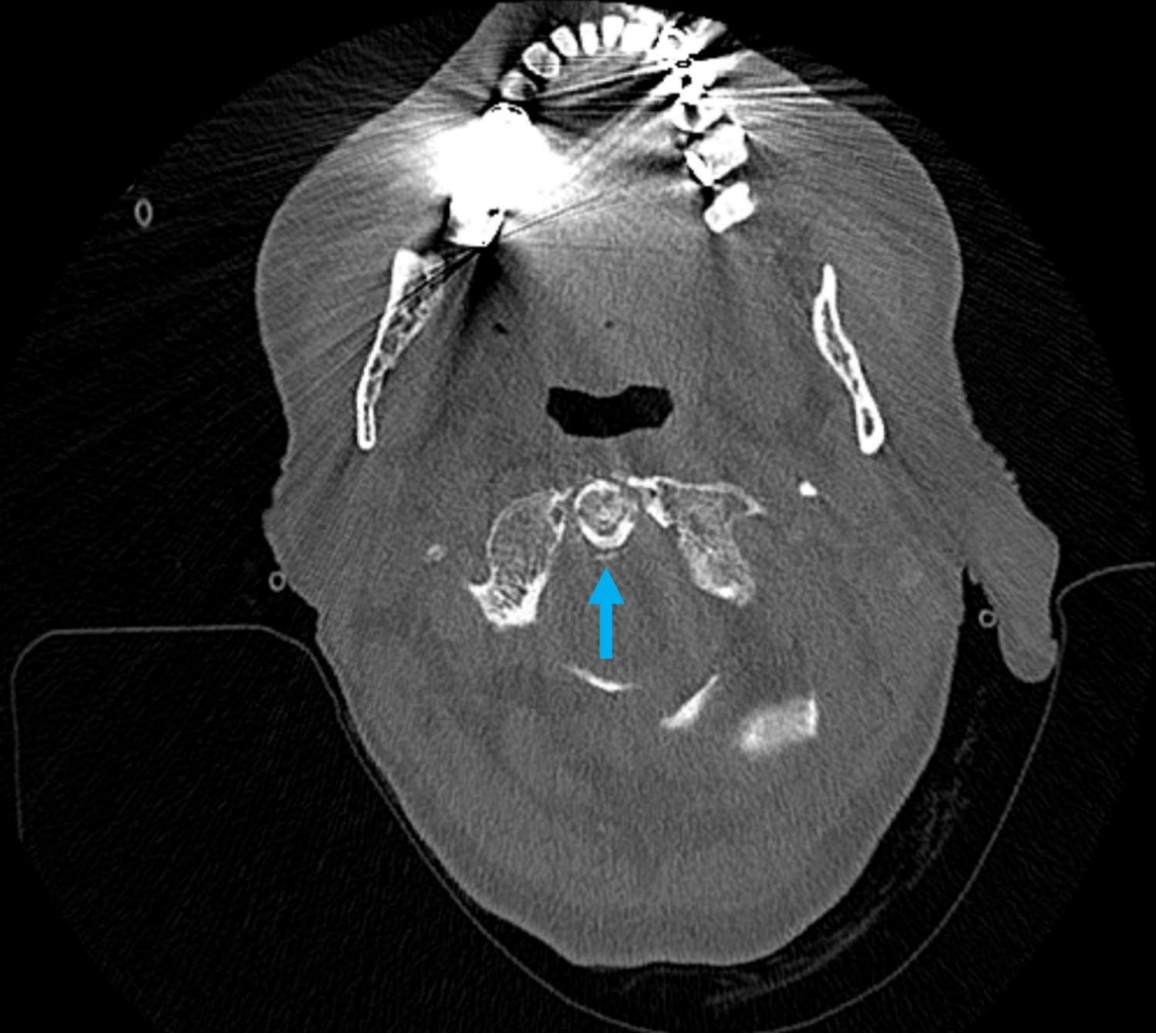
Conventional CT-scan of the cervical spine (axial plane). The arrow indicates typical ligamentous depositions around the odontoid process

To the Editor,

We report the case of an 84-year-old female who presented herself to the emergency department with general weakness, chills, cephalgia and neck pain. Initial vital signs were remarkable for an elevated heart (102/min) and respiratory rate (20/min) and fever (38.1 °C). The onset of symptoms was 1 day prior to admission. The physical examination revealed a marked neck stiffness with an inability to rotate the head or bend it forward. Blood tests were remarkable for elevated levels of C-reactive protein (156.6 mg/l, reference range < 5 mg/l), lactate (37 mg/dl, reference range 5–20 mg/dl) and leukocytes (20.68/nl, reference range 3.9–10.5/nl). A lumbar puncture yielded values within the normal range for cell count, lactate and glucose in the cerebrospinal fluid. A computed tomography of the cervical spine was performed and showed signs typical of crowned dens syndrome (Fig. 1).

Crowned dens syndrome is a rare manifestation of calcium pyrophosphate dihydrate disease [1]. Crystal depositions and consecutive inflammation in the ligaments around the dens axis lead to neck stiffness, fever, and elevated inflammatory parameters [2]. Crowned dens syndrome may be considered in elderly patients who show typical signs and symptoms of meningitis if an analysis of the cerebrospinal fluid does not show changes typically associated with meningitis. The diagnosis may be missed if head imaging does not include the atlanto-axial joint. Treatment options include non-steroidal anti-inflammatory drugs, steroids, colchicine and IL-1 inhibition [3, 4]. As also observed in the case at hand, most patients’ symptoms resolve quickly after treatment initiation.
